# Selective Detection of Lysozyme Biomarker Utilizing Large Area Chemical Vapor Deposition-Grown Graphene-Based Field-Effect Transistor

**DOI:** 10.3389/fbioe.2018.00029

**Published:** 2018-03-22

**Authors:** Sujoy Ghosh, Niazul I. Khan, John G. Tsavalas, Edward Song

**Affiliations:** ^1^Department of Electrical and Computer Engineering, University of New Hampshire, Durham, NH, United States; ^2^Center for Advanced Materials and Manufacturing Innovation, University of New Hampshire, Durham, NH, United States; ^3^Department of Chemistry, University of New Hampshire, Durham, NH, United States

**Keywords:** graphene, field-effect transistor, biosensor, aptamer, charge neutrality point, lysozyme, protein biomarker

## Abstract

Selective and rapid detection of biomarkers is of utmost importance in modern day health care for early stage diagnosis to prevent fatal diseases and infections. Among several protein biomarkers, the role of lysozyme has been found to be especially important in human immune system to prevent several bacterial infections and other chronic disease such as bronchopulmonary dysplasia. Thus, real-time monitoring of lysozyme concentration in a human body can pave a facile route for early warning for potential bacterial infections. Here, we present for the first time a label-free lysozyme protein sensor that is rapid and selective based on a graphene field-effect transistor (GFET) functionalized with selectively designed single-stranded probe DNA (pDNA) with high binding affinity toward lysozyme molecules. When the target lysozyme molecules bind to the surface-immobilized pDNAs, the resulting shift of the charge neutrality points of the GFET device, also known as the Dirac voltage, varied systematically with the concentration of target lysozyme molecules. The experimental results show that the GFET-based biosensor is capable of detecting lysozyme molecules in the concentration range from 10 nM to 1 µM.

## Introduction

Lysozyme is a ubiquitous enzyme that is widely available in diverse organisms, such as bacteria, bacteriophages, fungi, plants, and mammals. Being an antimicrobial protein, lysozyme is often called the “body’s own antibiotic” (Cheng et al., 2007; Lian et al., 2014). The protein is also extensively exploited in food industries for several purposes such as preserving meat and dairy products, as well as fruits and vegetables. The molecular weight of lysozyme is 14,400 Da with a primary sequence containing 129 amino acids, and it has an isoelectric point of 11.0 that causes lysozyme to behave as positively charged at neutral pH (Cheng et al., 2007). In addition to its extensive use in food industry, lysozyme also plays a vital role as a biomarker for diagnosing various diseases such as breast cancer (Serra et al., 2002), Alzheimer’s (Sandin et al., 2015), and rheumatoid arthritis (Torsteinsdóttir et al., 1999).

In the past, several biosensing techniques have been deployed for effective detection of lysozyme molecules. Some of these methods include chromatographic or antibody-based techniques (Ocaña et al., 2015), sensitive colorimetric detection (Huang et al., 2012), surface plasmon resonance-based approach (Subramanian et al., 2013), and electrochemical impedance spectroscopy measurement (Rodríguez and Rivas, 2009; Chen and Guo, 2013), to name a few. Among these sensing techniques, field-effect transistor (FET)-based sensing offers several advantages including miniaturization, low cost, and large-scale integration with other sensors as well as rapid detection and high sensitivity (Niwa et al., 2005; Wang et al., 2009; Hideshima et al., 2011).

A typical FET biosensor is comprised of a semiconducting channel contacted between the source and the drain electrodes. Upon adsorption of the biomolecules on the semiconductor surface, a change in the electric field occurs which affects the gate potential of the device resulting in a change in the charge carrier density within the channel of the FET. Such change in the drain current can be conveniently measured and be utilized as an interrogation strategy to probe the adsorbed biomolecules. This type of sensing mechanism has been demonstrated in the past for detecting target analytes in gases, water as well as in human serum (Lu et al., 2009; Huang et al., 2013; Zhou et al., 2014; Mao and Chen, 2017; Mao et al., 2017). Two-dimensional (2D) nanomaterials such as graphene, MoS_2_, WS_2_, etc., are particularly attractive as a channel material for FET-based biosensors due to their planner structure, excellent electrical properties and high surface area-to-volume ratio. Among several 2D materials graphene has been widely used as a promising FET channel material for various analyte detection due to its superior physical and chemical properties: namely, high intrinsic carrier mobility, good biocompatibility, high stability, and flexibility, which are all desirable traits to have for biosensing applications. For example, chemical vapor deposition (CVD)-grown graphene field-effect transistor (GFET) biosensors have been used to detect triphosphate (Xu et al., 2014) and binding kinetics of DNA hybridization (Xu et al., 2017). Similarly, Huang et al. (2011) and Chen et al. (2017) have successfully demonstrated the detection of bacteria and Ebola antigen using graphene-based FETs. Nonetheless, the detection of protein molecules using FET biosensors is largely limited by the charge screening effects of the non-specifically adsorbed surface molecules from the buffer solution. To overcome this issue, the graphene channel surface is typically modified with target receptors which enable specific binding reaction with the charged target protein molecules in the solution. For example, Ohno et al. (2011) reported that in an aptamer-modified GFET, a non-specific binding of the non-target protein molecules was suppressed. However, this technique is still limited for specific detection of small and weakly charged analytes which do not directly induce detectable changes in surface charge after molecular binding. Moreover, the detection of lysozyme protein *via* a GFET-based biosensing platform has not yet been demonstrated so far. Therefore, in this work, we describe the selective detection of lysozyme molecules utilizing large area CVD-grown GFET devices prepared by a facile one step transfer process.

**Figure 1:**
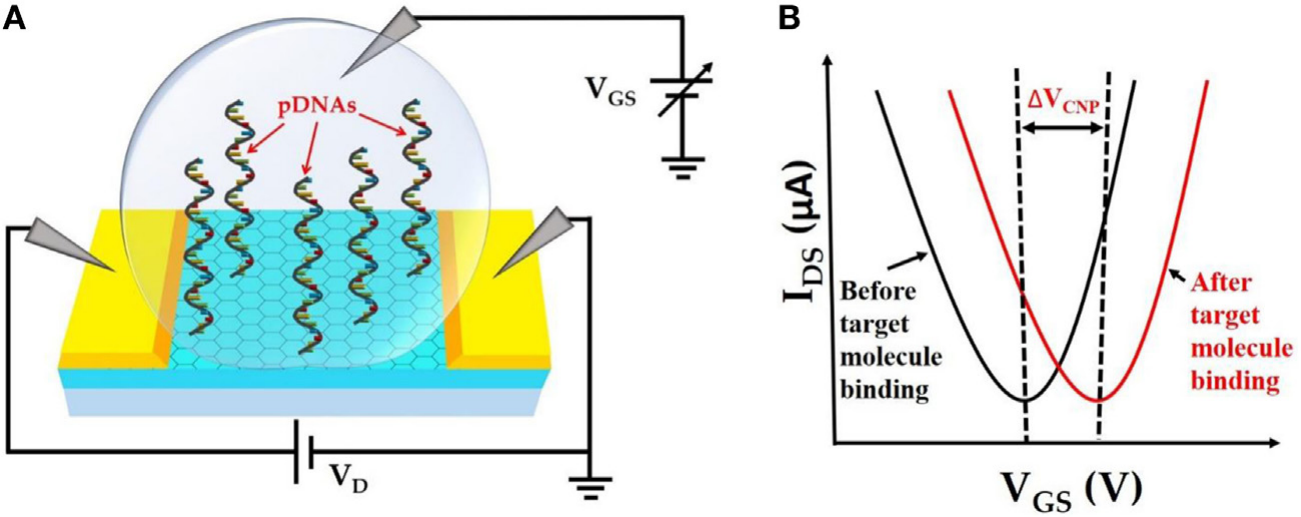
**(A)**. Schematic representation of top liquid-gated graphene field-effect transistor (GFET) device with anchored probe DNAs on the graphene channel surface. **(B)** *I*_DS_–*V*_GS_ characteristics of GFET device before and after target molecule binding resulting in a detectable change in *V*_CNP_.

The fundamental operating principle of the GFET biosensor is illustrated in Figure 1. Figure 1A depicts the schematic of the liquid-gated GFET device. CVD-grown large area graphene is contacted with source and drain electrodes. Single-stranded probe DNAs (pDNAs), which act as target-binding aptamers, are securely anchored onto the graphene surface, *via* the bifunctional linker 1-pyrenebutyric acid *N*-hydroxysuccinimide ester (PBASE). A sample ionic buffer solution is dropped on the surface of the GFET channel. Upon applying a gate voltage (*V*_GS_), between the gate electrode in the solution and the source electrode of the GFET channel, the electrical double layer (EDL) is formed at the interface between the graphene channel and the electrolyte (Xia et al., 2009). This formation of EDL induces image charges in the channel and provides high gate capacitance. This gating capacitance provides the source of electrostatic gating of the GFET. Figure 1B demonstrates the *I*_DS_–*V*_GS_ characteristics of the GFET. A typical ambipolar electric field-effect characteristic is expected for the top-gate operation with −1 ≤ *V*_GS_ ≤ 1 V. The minimum *I*_DS_ occur at the charge neutrality point *V*_CNP_ also known as the Dirac voltage (*V*_Dirac_), which signifies the demarcation between the *p*-type and the *n*-type conduction of the graphene channel. Therefore, the *V*_CNP_ represents the doping level in the graphene channel. Since the surface–analyte or analyte–analyte bindings occur in the proximity of the graphene surface, the analyte–analyte bindings can significantly change the doping level in the graphene channel. This change in the doping level results in a detectable shift in *V*_CNP_ as shown in Figure 1B.

## Materials and Methods

### Materials

The amino linker modified anti-lysozyme DNA oligonucleotide [sequence designed by Cox and Ellington (2001)] was synthesized by Sigma-Aldrich. The sequence of the oligonucleotide is: 5′-amino-C6-ATC AGG GCT AAA GAG TGC AGA GTT ACT TAG-3′. Lysozyme protein from chicken egg white was also purchased from Sigma-Aldrich. Protein stock solutions were prepared by dissolving the lyophilites in fresh ultrapure triple-distilled deionized water and stored at −20°C. The diluted solutions of proteins were prepared in 0.1 mM Phosphate-buffered solution (PBS, pH 7.4). PBS was obtained from Sigma-Aldrich. Tween 20 and 1-pyrenebutyric acid *N*-hydroxysuccinimide ester (PBASE) were purchased from RPI Research Products International (IL, USA) and Santa Cruz Biotechnology (TX, USA), respectively.

### Fabrication of GFET

**Figure 2:**
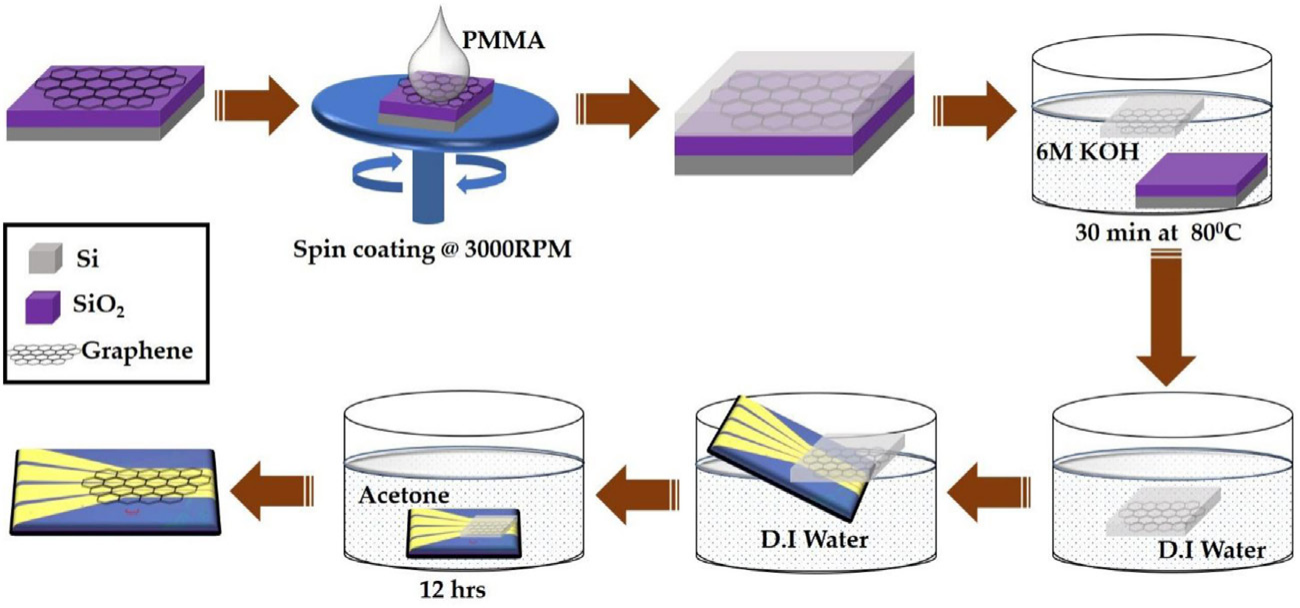
Schematic illustration of large area chemical vapor deposition-graphene field-effect transistor device fabrication process.

Figure 2 shows the transfer process of large area CVD-grown graphene from SiO_2_/Si substrate onto the prefabricated four independently addressable gold electrodes. The CVD-grown graphene sample was purchased from Graphene Supermarket (NY, USA). The transfer process begins with spin coating onto the graphene a support layer of poly (methyl methacrylate) (PMMA) at 3,000 RPM followed by immersion into 6 M KOH solution for 30 min at 80°C. This results in etching of the underlying SiO_2_ layer and separation of the top PMMA/graphene bilayer from the substrate. The PMMA-protected graphene layer was then collected on top of the prefabricated gold electrodes and dried at room temperature. The electrodes were then immersed into acetone for 12 h to dissolve the top PMMA layer followed by consecutive washing with ethanol and DI water. Finally, the devices were annealed at 250°C for 2 h in an argon-filled chamber to reduce any PMMA residues (Lerner et al., 2014).

### Electrical FET Measurements

All electrical measurements were carried out using the Keysight precision source/measure unit (B2902A) combined with a probe system (Micromanipulator: 450PM-B). For FET measurements, solution-gate experiments were performed. A constant bias voltage *V*_DS_ = 100 mV was applied across the drain and the source terminals by connecting the two manipulator needles to the source and the drain electrodes. The gate voltage *V*_GS_ (−1 ≤ *V*_GS_ ≤ +1 V) is applied by immersing the third manipulator needle into the sample droplet of 0.01× PBS buffer solution placed on top of the GFET devices.

### Functionalization of GFET

Immobilization of the probe DNAs (pDNAs) onto the graphene surface was performed by incubating the graphene chip in the bifunctional linker 1-pyrenebutyric acid *N*-hydroxysuccinimide ester (PBASE) at 10 mM in dimethyl formamide (DMF) at room temperature for 20 h. The aromatic pyrenyl group of PBASE binds to the basal plane of graphene through non-covalent π–π interactions (Chen et al., 2001). This was then followed by rinsing the chip sequentially in DMF, ethanol and DI water for 3 min each. In the final step, the chip was incubated with the aminated (5′) pDNA at 5 µM in 0.01× PBS at room temperature for 12 h to covalently link the pDNA to the PBASE *via* an *N*-hydroxysuccinimide cross-linking reaction (Hermanson, 2013; Gao et al., 2016). To remove the unanchored pDNAs, the chip was successively rinsed with 0.01× PBS and DI water. Following the probe attachment, the chip was treated with 0.1% Tween 20 followed by sequential rinsing in 0.05% Tween 20 and DI water. Finally, the chip was incubated in different concentrations of target proteins in 0.01× PBS for 30 min. This allows lysozyme binding due to the sequence-specific high affinity of the aptamers to lysozyme (Rohrbach et al., 2012; Khan et al., 2018). Afterward, the chip was rinsed with 0.01× PBS buffer followed by DI water and dried with a compressed air gun before performing the electrical measurements.

## Results and Discussion

### The Effects of Functionalization and DNA Immobilization on the FET Measurements

For the selective protein detection, the graphene layer is successively functionalized by PBASE and the single-stranded pDNAs specifically designed for lysozyme binding (Cox and Ellington, 2001). The GFET devices were configured as electrolyte-gated FETs where the graphene is the conducting channel formed between the source and the drain electrodes on the SiO_2_/Si substrate as schematically depicted in Figure 1A. PBS solution (0.01×) was used as the top gating dielectric. The pyrene group terminated PBASE is coupled to the graphene surface *via* the π–π stacking forces (Xu et al., 2017). The 5′-amino-modified pDNAs were attached to the amine-reactive succinimide group of PBASE by the conjugation reaction between the amine groups. The *I*_DS_–*V*_GS_ characteristics of the GFET devices were measured sequentially after each functionalization step and exposure to the target lysozyme molecules. The binding of the lysozyme molecules to the pDNAs induces changes in the charge carrier density in the graphene channel. This causes a detectable change in the Dirac voltage (*V*_Dirac_) or the charge neutrality point (*V*_CNP_) in the *I*_DS_–*V*_GS_ characteristics of the GFET.

**Figure 3:**
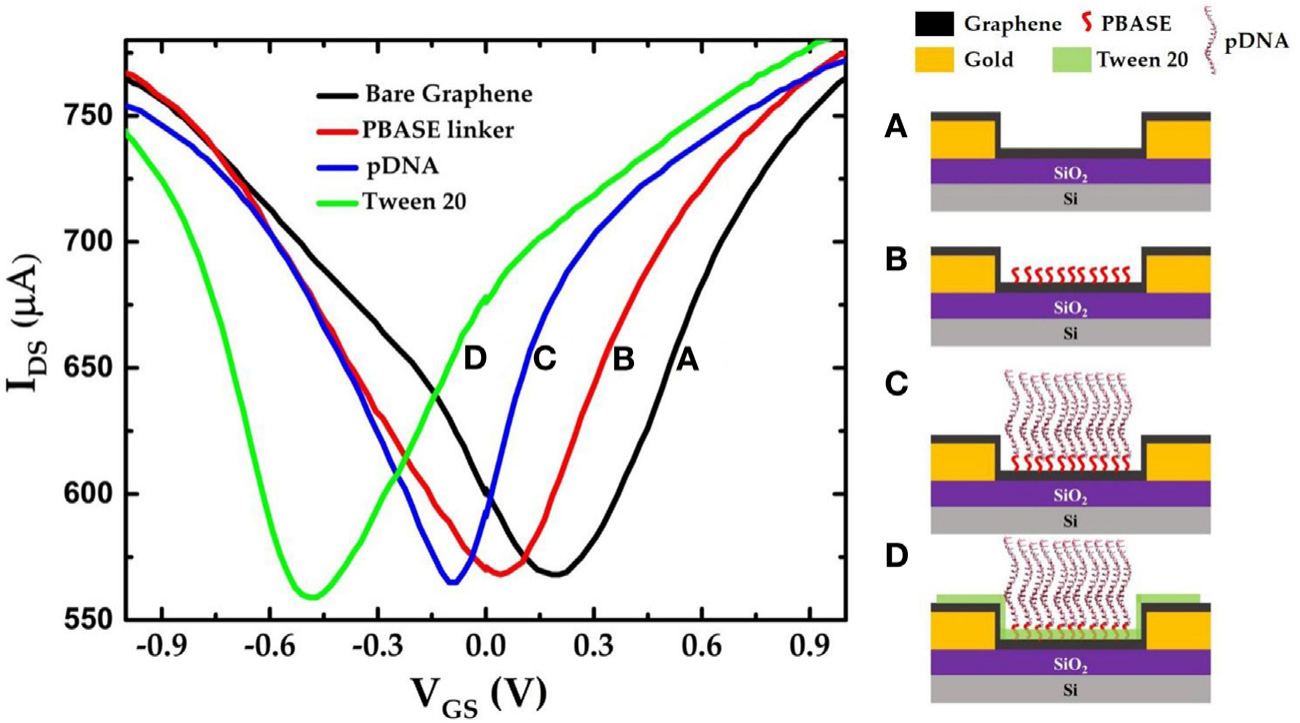
*I*_DS_–*V*_GS_ characteristics of the graphene field-effect transistor device (a) before any surface modification (unmodified graphene); (b) after PBASE functionalization; (c) after attaching single-strand probe DNAs (pDNAs) to the PBASE linker; and (d) after treating the graphene surface with 0.1% Tween 20.

Figure 3 shows the *I*_DS_–*V*_GS_ characteristics of a GFET device at each stage during the surface modification process. The *I*_DS_–*V*_GS_ characteristics exhibit ambipolar behavior as the gate voltage in the top-gate dielectric (0.01× PBS) changes from −1 to +1 V similar to previously reported measurements (Xu et al., 2017). The *V*_CNP_ for the unmodified GFET was found to be 203.96 mV. Since the graphene channel is sensitive to any surface adsorptions or modifications, the *V*_CNP_ was shifted left at 40.8 mV relative to the unmodified graphene channel after the PBASE linker modification. Previously, Wu et al. reported that PBASE modification of graphene causes n-doping in the graphene channel after long incubation in the DMF solvent (Wu et al., 2017). Therefore, left shift of *V*_CNP_ in our experimental results suggests *n*-doping of the graphene channel. Figure 3C shows the *I*_DS_–*V*_GS_ characteristics of the GFET after the pDNA attachment. Here, we note that the *V*_CNP_ further shifted left with respect to that after PBASE modification (Figure 3B) indicating further *n*-doping of the graphene channel. It has been widely observed and speculated that the presence of electron rich nucleotide bases in the DNA molecules can cause n-doping effects in carbon nanotubes and graphene (Gui et al., 2006; Dong et al., 2010). We have further treated the GFET devices with 0.1% Tween 20 solution in deionized water to minimize non-specific adsorption. Due to its high affinity with graphene, Tween 20 has been extensively used in the past to deter non-specific binding of proteins as well as to remove non-specifically adsorbed pDNAs on the graphene (Gao et al., 2016). However, the presence of the surfactant adsorbates can effectively dope the graphene channel. Among various surfactants, Tween 20 has been reported to cause n-doping effect on the graphene (Shih et al., 2012). Further negative shift of *V*_CNP_ in the *I*_DS_–*V*_GS_ curve after Tween 20 treatment thus is consistent with an increased n-doping effect as indicated in Figure 3D. We further notice a small change in the minimum current at *V*_CNP_ that after each step of functionalization. Due to atomically thin nature, the minimum conductance at the charge neutrality point *V*_CNP_ in GFET devices are extremely sensitive to several extrinsic factors such as charge impurities, doping density, external ions, etc. (Tan et al., 2007; Chen et al., 2008). Previously it was also reported that the minimum conductance can also be affected by the presence of PBS buffer ions (Dong et al., 2010). Thus, we believe that the small changes in the minimum current at *V*_CNP_ in our GFET devices are caused due to doping effect after surface modification and/or due to the ionic adsorption or desorption effects of the PBS buffer ions.

### Concentration Dependent Shift in the Charge Neutrality Point

**Figure 4:**
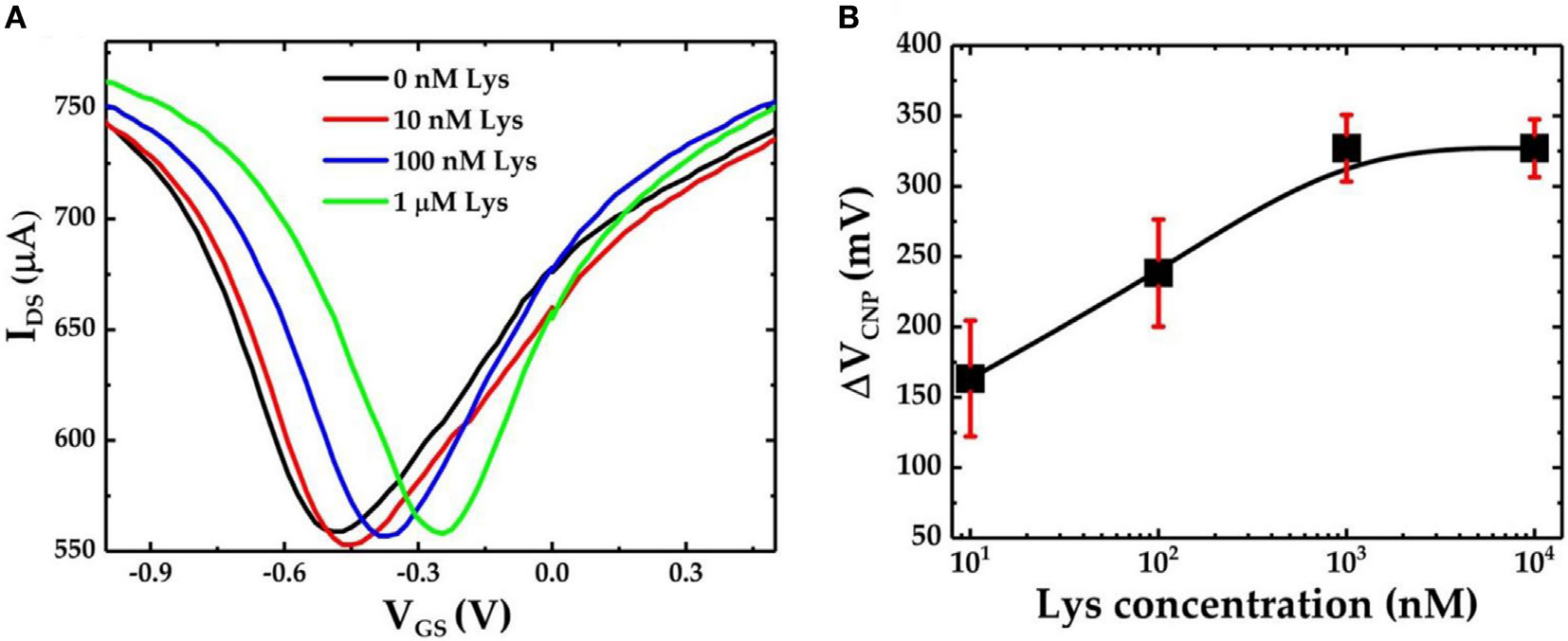
**(A)** *I*_DS_–*V*_GS_ characteristics of the graphene field-effect transistor (GFET)-based biosensor device when it is exposed to varying concentrations of lysozyme protein and **(B)** the calibration curve for the GFET-based biosensor showing Δ*V*_CNP_ as a function of different concentrations of lysozyme. The sample set is *n* = 3, and the error bar represents 1 SE.

Figure 4A shows the *I*_DS_–*V*_GS_ characteristics of the GFET device when exposed to varying concentrations of lysozyme samples. The graphene devices were first incubated in 0.01× PBS buffer solution containing the lysozyme protein for 30 min followed by a gentle wash in PBS and deionized water before the FET measurements were performed. We found that after exposure to 10 nM lysozyme solution the *V*_CNP_ shifted to −449 mV. This results in a positive shift of *V*_CNP_ of 20.5 mV with respect to the *V*_CNP_ = −469.5 mV at 0 nM lysozyme. *V*_CNP_ shifts further right with the increasing lysozyme concentration. The lysozyme binding with the pDNA aptamer (5′-amino-C6-ATC AGG GCT AAA GAG TGC AGA GTT ACT TAG-3′) was previously confirmed by Cheng et al. (2007). It was also found that at neutral pH, lysozyme is positively charged with net +8 charges (Blake et al., 1965; Cheng et al., 2007). Therefore, the presence of lysozyme molecules in the proximity of the graphene nanosheet can induce a p-doping effect in the FET channel. Thus, the positive shift of the *V*_CNP_ can be attributed to the reduction of n-doping effects during the previous functionalization steps. Furthermore, our results suggest a strong correlation between the lysozyme concentration and the degree of the *V*_CNP_ shift in the right direction: the higher the lysozyme concentration, the further the *V*_CNP_ shifts to the right. Figure 4B shows the relative shift of *V*_CNP_ (Δ*V*_CNP_) (with respect to the position of *V*_CNP_ after exposure to 0 nM lysozyme concentration) after exposing the GFET devices to a series of lysozyme concentrations in the range from 10 nM to 10 µM. From the FET responses, we have found that Δ*V*_CNP_ increases sharply for the lower concentrations of lysozyme and then gradually reaches saturation at approximately 1 µM and beyond.

**Figure 5:**
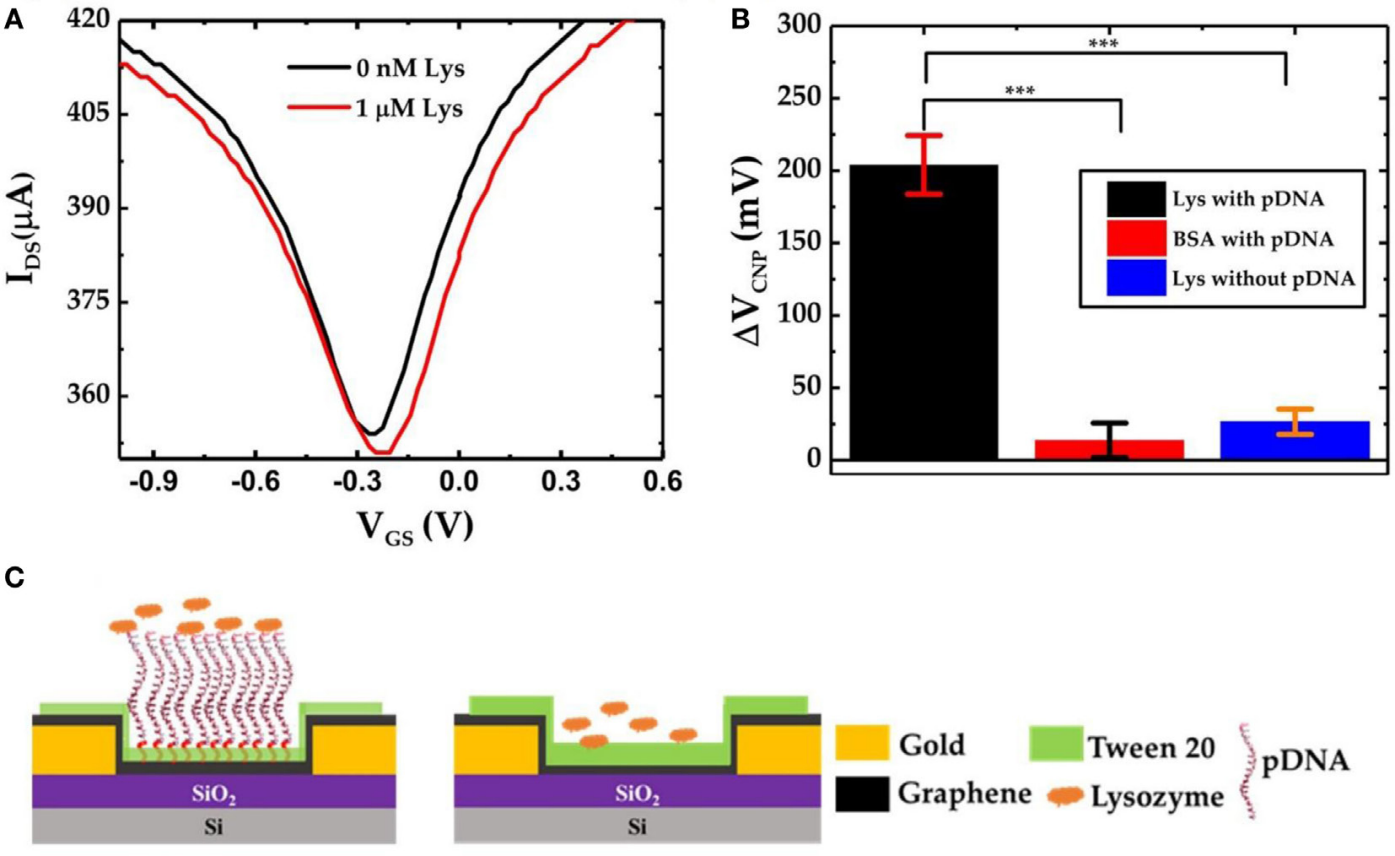
**(A)** *I*_DS_–*V*_GS_ characteristics of graphene-PBASE field-effect transistor (FET) [without probe DNA (pDNA)] device before and after exposure to 1 µM lysozyme; **(B)** comparative bar chart showing the Δ*V*_CNP_ of the graphene-PBASE FET devices with the pDNA functionalization after exposure to 1 µM lysozyme and 1 µM bovine serum albumin and without pDNA functionalization (*n* = 3, error bar = 1 SD, paired Student’s *t*-test, ***p < 0.001); and **(C)** the schematic diagram of the graphene field-effect transistor with pDNAs (left) and without pDNAs (right).

To further verify the specific lysozyme binding with the pDNA aptamers and subsequently to characterize the selectivity of the GFET biosensor devices, we also prepared GFET devices but without the presence of pDNAs as shown in Figure 5C. After successive functionalization with PBASE linker and 0.1% Tween 20, the devices were exposed to 1 µM lysozyme solution. The *I*_DS_–*V*_GS_ curves obtained from the GFET without pDNAs are shown in Figure 5A. Here we found that, after exposure to the lysozyme molecules, there is only a very small shift in *V*_CNP_ (Δ*V*_CNP_ = 10 mV). This slight change in *V*_CNP_ can be attributed to the small amounts of non-specific surface adsorptions of the lysozyme proteins on the surface of the graphene sheet. Similarly, we tested pDNA functionalized GFET devices against another non-specific target protein bovine serum albumin (BSA). As expected, due to the lower binding affinity of the pDNA aptamers with BSA, negligible changes in *V*_CNP_ were observed. Figure 5B compares the overall sensor responses of the three GFETs, two with the pDNA modification against lysozyme and BSA and one without the presence of pDNAs against the lysozyme (three separate devices in each group). These results clearly indicate that our graphene-pDNA FET devices can selectively detect lysozyme molecules with significant changes in the charge neutrality point.

## Conclusion

We have presented aptamer-modified large area CVD-grown graphene-FET biosensor for the detection of lysozyme protein biomarker. The FET biosensor is sequentially functionalized with PBASE crosslinker, an aptamer specifically designed for the molecular recognition of lysozyme protein and Tween 20 as a blocking agent for minimizing non-specific adsorptions on the graphene channel surface. We have demonstrated that the lysozyme molecules have specifically bound to the surface-immobilized aptamers causing a disruption in the charge carrier density. This resulted in the shifting of the charge neutrality point. Consequently, this change in the charge neutrality point potential of the graphene-FET devices was utilized to quantify the bound lysozyme concentration. The graphene-FET biosensor devices were tested for the detection of the lysozyme biomarker with concentrations ranging from 10 nM to 1 µM in the PBS buffer, demonstrating its capability as a specific biomarker sensor. Furthermore, the dynamic drain-source current measurement with respect to varying lysozyme concentrations would be essential for the demonstration of real-time monitoring of lysozyme molecules. In terms of health diagnostics application, this technology can potentially be used for facile development of large-scale point-of-care testing kits for low-cost and fast-readout disease screening and diagnostics.

## Author Contributions

SG and ES conceived the GFET-based biosensing of lysozyme protein; SG and NK performed the device fabrication, collected the experimental data, and wrote the manuscript; SG, NK, JT, and ES performed data analysis; ES and JT oversaw the project and performed the overall editing of the manuscript.

## Conflict of Interest Statement

The authors declare that the research was conducted in the absence of any commercial or financial relationships that could be construed as a potential conflict of interest.

## Supplementary Material

Confirmation of lysozyme binding to the aptamer sequence by using electrochemical square wave voltammetry.
