# Supplementary material for: Selective Detection of Lysozyme Biomarker Utilizing Large Area Chemical Vapor Deposition-Grown Graphene-Based Field-Effect Transistor
Source: Front Bioeng Biotechnol. 2018 Mar 22;6:29. doi: 10.3389/fbioe.2018.00029 (PMC5890177; doi:10.3389/fbioe.2018.00029)
Supplement: Supplementary file 1 — Confirmation of lysozyme binding to the aptamer sequence by using electrochemical square wave voltammetry. [file Data_Sheet_1.docx]

Supplementary Material

Selective Detection of Lysozyme Biomarker Utilizing Large Area CVD Grown Graphene Based Field Effect Transistor

Sujoy Ghosh^1,3^, Niazul I. Khan^1^, John G. Tsavalas^2,3^, Edward Song^1,3*^

^1^Department of Electrical and Computer Engineering, University of New Hampshire, Durham, NH, USA

^2^Department of Chemistry, University of New Hampshire, Durham, NH, USA

^3^Center for Advanced Materials and Manufacturing Innovation, University of New Hampshire, Durham, NH, USA

# Lysozyme Binding Confirmation

Lysozyme binding to the aptamers was confirmed by performing experiments with methylene blue (MB)-labelled thiolated DNA aptamers (LGC Biosearch Technologies, CA, USA) with the same sequence as used by Ellington *et al*. (**Cox and Ellington 2001**) on gold rod electrode. The DNA-based electrochemical lysozyme sensor was fabricated on a gold rod electrode (A-002421, Bio-Logic USA Science Instruments, TN, USA) of 3 mm diameter using a previously described method (**Xiao, Lai, and Plaxco 2007**). The experiments were performed in a 3-electrode electrochemical cell with Ag/AgCl as the reference electrode and platinum as the counter electrode. The results are presented in Supplementary Figure 1.


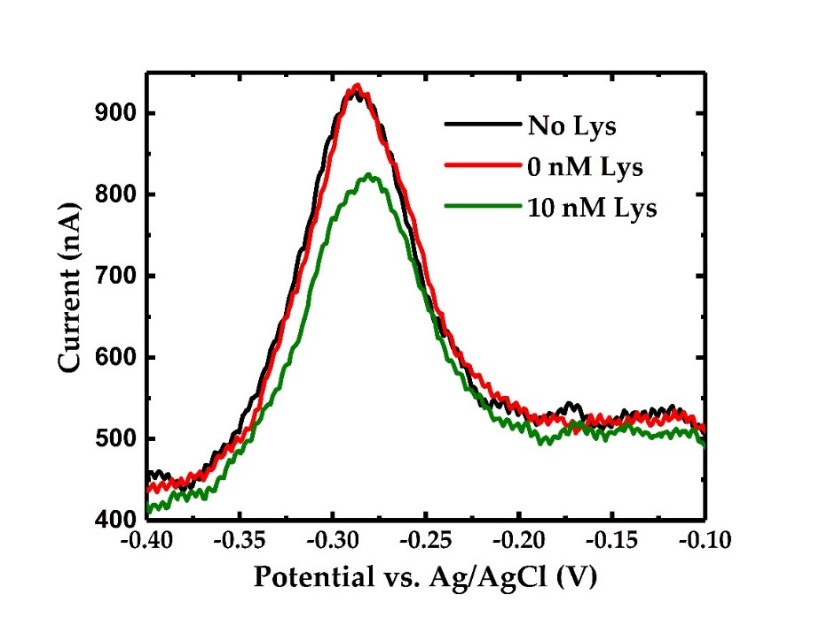


**Supplementary Figure 1.** Square wave voltammograms obtained for different concentrations of lysozyme in 0.01X PBS.

It can be seen from Supplementary Figure 1 that the peak current reduces when lysozyme is exposed to the aptamer-modified gold electrode which suggests that specific binding of lysozyme to the aptamer-based recognition element has occurred (**Xiao et al. 2005; Macazo, Karpel, and White 2015**). In the absence of lysozyme, the MB-labels are easily accessible to the electrode which enables efficient electron transfer between the MB and the electrode. This causes high voltammetric peak current for the reduction of MB as characterized using square wave voltammetry (SWV). When lysozyme comes in proximity to the aptamers, due to the specific affinity the aptamers undergoe conformational change to capture the proteins. This alters the electron tunneling distance hindering the charge transfer from the MB to the electrode. As a result, the voltammetric peak current decreases.

# References

Cox, J. C., and A. D. Ellington. 2001. “Automated Selection of Anti-Protein Aptamers.” *Bioorganic & Medicinal Chemistry* 9 (10):2525–31.

Macazo, Florika C., Richard L. Karpel, and Ryan J. White. 2015. “Monitoring Cooperative Binding Using Electrochemical DNA-Based Sensors.” *Langmuir* 31 (2):868–75. https://doi.org/10.1021/la504083c.

Xiao, Yi, Rebecca Y. Lai, and Kevin W. Plaxco. 2007. “Preparation of Electrode-Immobilized, Redox-Modified Oligonucleotides for Electrochemical DNA and Aptamer-Based Sensing.” *Nature Protocols* 2 (11):2875–80. https://doi.org/10.1038/nprot.2007.413.

Xiao, Yi, Arica A. Lubin, Alan J. Heeger, and Kevin W. Plaxco. 2005. “Label-Free Electronic Detection of Thrombin in Blood Serum by Using an Aptamer-Based Sensor.” *Angewandte Chemie (International Ed. in English)* 44 (34):5456–59. https://doi.org/10.1002/anie.200500989.
